# Self-efficacy and resilience as predictors of students’ academic motivation in online education

**DOI:** 10.1371/journal.pone.0285984

**Published:** 2023-05-23

**Authors:** Parisa Abdolrezapour, Sahar Jahanbakhsh Ganjeh, Nasim Ghanbari

**Affiliations:** 1 Department of English, Salman Farsi University of Kazerun, Kazerun, Iran; 2 Department of Psychology, Yasouj University, Yasouj, Iran; 3 Department of English Language and Literature, Faculty of Humanities, Persian Gulf University, Bushehr, Iran; Ahvaz Jundishapur University: Ahvaz Jondishapour University of Medical Sciences, ISLAMIC REPUBLIC OF IRAN

## Abstract

Motivation as a catalyst for human conduct has been associated with lots of variables. However, self-efficacy and resilience as two important components of the individuals’ psychological capital have not received enough scientific attention. This gets more significance considering the global COVID-19 pandemic with noticeable psychological consequences for the learners receiving online education. Hence, the current study proceeded to investigate the relationship between students’ self-efficacy, resilience, and academic motivation in online education. To this aim, a convenience sample of 120 university students coming from two state universities in south of Iran participated in an online survey. The questionnaires used in the survey included the self-efficacy questionnaire, resilience questionnaire, and academic motivation questionnaire. Pearson correlation and multiple regression statistical methods were applied to analyze the obtained data. The results pointed to a positive relationship between self-efficacy and academic motivation. In addition, those with a higher degree of resilience were found to experience higher academic motivation. Moreover, the results of multiple regression test revealed that self-efficacy and resilience can significantly predict the academic motivation of the students involved in an online mode of education. The research proposes a number of recommendations for developing the learners’ level of self-efficacy and resilience through enacting various pedagogical interventions. In this way, a heightened level of academic motivation would enhance EFL learners’ learning rate.

## Introduction

The COVID-19 outbreak made an abrupt transition to online platforms for many businesses and schools were no exception. This transition brought new challenges for individuals and profoundly affected their perceptions, feelings and attitudes [1, 2]. They mostly found it difficult to overcome their stress and maintain their motivation, despite the persistence of quarantine conditions and the ever-increasing concerns about the virus spread. Such ever-growing stress did also affect university students and their academic performance and beliefs as they have experienced dramatic changes in habits and their daily routines [3].

The research to date has confirmed learners’ challenges and psychological problems caused by the coronavirus pandemic. In this regard, a number of studies were conducted to investigate such factors as learners’ mental health [4, 5], learners’ attitude [6], motivation [7], anxiety and coping strategies [8], as well as learners’ [1] and teachers’ perceived flow [2]. However, there has been no reliable evidence on the contribution of two highly researched psychological capital variables (i.e., self-efficacy and resilience) in traditional classes for the academic motivation of the students involved in online education. Therefore, this paper was set out to address this gap in the literature.

## Academic motivation

Motivation has received increasing attention in a wide range of disciplines. It is regarded as a multidimensional construct encompassing various constituents as goals, beliefs, perceptions, values, emotions, and needs [9–11]. As Gottfried [12] states, academic motivation refers to the “enjoyment of school learning characterized by a mastery orientation, curiosity, persistence, task-endogeny, and the learning of challenging, difficult, and novel tasks” (p.525).

This study applies one of the most prominent theories in research on motivation, i.e., self-determination theory [13–15], which is also a macro-theory of human development and well-being and has been extensively applied in education. This theory makes a distinction between autonomous (intrinsic) and controlled (extrinsic) motivation that explains why individuals act in a certain way. According to Ryan and Deci [16], the key determinants of academic motivation are the fulfillment of the needs for autonomy, relatedness, and competence. However, these conditions might not be met in an online education context. As for autonomy, teachers find it difficult to cultivate learners’ autonomy by allowing them to have a choice and most students do not possess the independent learning skills; students struggle to measure their competence when there is no interaction with the environment and the peers and little formative feedback has been provided; and finally, when working from home and following the lectures alone, students’ perception of social group would be transformed and there would be lower emotional bond among the peers [17]. Despite the learners’ higher motivation in some online classes [18], which might be due to the choice they have been awarded, there has been a decrease in the level of their motivation amidst the coronavirus pandemic [19]. Given the importance of academic motivation in students’ cognitive engagement in the academic tasks, several researchers focused on the influence of the COVID-19 restrictions on students’ academic motivation [19, 20] and they confirmed the significant effect of the pandemic in decreasing learners’ motivation. In addition, this transition was associated with increased negative emotions such as stress and anxiety [8, 21, 22]. Previous studies also confirmed the role of self-efficacy in individuals’ level of stress [23]. Thus, it can be hypothesized that those with a lower level of control on self-efficacy would experience higher stress and subsequently lower motivation. However, there is no empirical study confirming this issue during the COVID-19 pandemic. Therefore, self-efficacy was considered as another variable in this study.

### Self-efficacy

Following Bandura’s [24] social cognitive theory, in this article, self-efficacy is defined as “beliefs in one’s capabilities to organize and execute the courses of action required to produce given attainments” (p.3), which has a significant contribution in one’s choice of activity and the time devoted to a given task [25]. This concept is associated with cognitive, motivational, and affective processes that would subsequently influence effective performance [26]. Bandura [27] situates self-efficacy within an expectancy-value framework, whereby “motivation is the product of the expectation that a given course of action will produce certain outcomes and the value placed on those outcomes” (p. 28). Therefore, self-determination theory (SDT), with its emphasis on intrinsic and extrinsic motivation, and expectancy-value framework, and in consequence self-efficacy theory, are clearly related.

Self-efficacy has been the focus of several second/foreign language researchers [28–33], all confirming its positive correlation with academic success and performance in different language subskills including speaking [34, 35], reading [36], listening [37, 38], and writing [39, 40]. In other words, the literature confirmed that a strong sense of self-efficacy could predict individuals’ better language performance and learning achievement.

Self-efficacy has also been found to be a significant factor in online education environments [41–43] due to learners’ new experiences associated with the online context. However, considering the stress associated with education during the pandemic, which is mainly caused by task overload, pandemic fears, social isolation, and confinement [44–46], it was hypothesized that there would be a decline in students’ self-efficacy, which was found to be linked to their performance and had an indirect impact on the academic motivation [47]. As Pajares [48] claims, efficacious learners persist longer when encountering difficulties. Thus, there is a need for studies unraveling EFL learners’ self-efficacy level and find its possible contribution to academic motivation in this pandemic.

### Resilience

Resilience is defined variously in different social contexts. In psychology, for example, it is defined as the strategies adopted by individuals to respond to a challenging event [49], while in education, it deals with the students’ ability to cope with adversities and succeed [50]. In general, the term refers to an effective mechanism for overcoming adverse situations [51]. Previous studies dealing with the main components of resilience pointed to multiple factors including empathy and sociability [52, 53] as well as persistence in spite of hardship or discouragement [54]. The existing literature also confirmed the positive relationship between the resilience and educational success.

According to SDT, the satisfaction of the three basic psychological needs, i.e., relatedness, autonomy and competence, improves well-being and strengthens inner resources related to resilience. Therefore, SDT and data from several sources [55, 56] establish the correlation between motivation and resilience. In general, resilience is crucial for emotional balance and social success [57]; it is also “a process of interactive adaptation that facilitates coping in the face of adversity” [58] and as [59] claim, it “is pivotal to cope with stress and vital to stay in balance” (p.12). Thus, it is effective for coping with stressful periods of uncertainty such as the one accompanied by the COVID-19 pandemic. Accordingly, a number of researchers have sought to determine the key role played by resilience in the university or academic context [60], in people’s psychological and social state [61] and its significant relationship with the ability to handle the stress created during the COVID-19 pandemic [62]. In addition, [63] pointed to some critical factors in building and inhibiting resilience including support, community, leadership, and planning at universities. The authors also confirmed the pivotal role of online and flexible learning for the resilience-building.

However, despite the importance of resilience and its significant effect on decreasing individuals’ stress and increasing their psychological well-being, it was not until the last decade that researchers considered resilience worthy of scholarly attention in the second language context. Recent studies [52, 64–67] focused on its relation to motivation and proficiency in language learning. Following this line of research, [51] pointed that this concept should be studied alongside other variables with which it interacts, and a number of studies confirmed its significant role in the ability to manage the stress during the coronavirus pandemic [68–70]. Hence, the present study aims to focus on its ability to predict the individual’s academic motivation in online education.

### The present study

Alongside what mentioned above, the present research intended to study the contribution of two psychological variables of self-efficacy and resilience for the academic motivation of the English language learners in online education. Relying on the above grounds, the following two hypotheses were pursued in this study:

- Hypothesis 1. Students’ self-efficacy predicts their academic motivation in online education.
- Hypothesis 2. Students’ resilience predicts their academic motivation in online education.

### Methodology

#### Ethics statement

All procedures performed in the study were in accordance with the ethical guidelines of the 1964 Helsinki declaration and clearance from the Committee on Research Involving Human Subjects of Yasuj University was obtained. Moreover, at the onset of the study, our participants were informed that the data of the study will be used only for research purposes and that all data will be used anonymously throughout the study.

#### Participants

A convenience sample of 120 Iranian EFL learners (age range = 19–26, M = 21 years old, SD = 1.3) from two different state universities in south of Iran participated in this study. There were both male and female students in the study (35 males and 85 females). Participants were recruited via social media platforms (e.g., WhatsApp). They were all Persian native speakers studying for the bachelor’s degree of TEFL (teaching English as a foreign language) and mostly had studied English for about eight to eleven years. After obtaining the written informed consent from each participant, they were asked to complete the online survey.

### Instruments

To address the purpose of the present study, a number of questionnaires were used, which were all in English. Participants were undergraduate students studying English and it was supposed that they know the meaning of the words; however, in the instruction section, they were asked to check the dictionary, whenever they had difficulty understanding the wording of an item.

### Questionnaire of English Self-Efficacy (QESE)

To assess the degree of EFL learners’ self-efficacy, the self-efficacy questionnaire adapted from [71] was used. This instrument was originally developed from interviews, observations and verbal protocols of Chinese learners of English in the United States. The questionnaire intends to gain more insights into subjects’ judgements on their capabilities to accomplish certain tasks with English as their foreign language. This 32-item scale yields scores on four aspects of self-efficacy including speaking, listening, reading, and writing (8 items for each skill). Participants were required to answer on a 7-point Likert scale, with 1 indicating ‘I cannot do it at all’ and 7 indicating ‘I can do it very well’. [72] checked the validity of the scale in a sample of English language learners and confirmed that it measures a unidimensional construct. Furthermore, its high reliability was reported by [71] with the person reliability of .99 and item reliability of .98. Also, in this research, the Cronbach’s alpha coefficient of the questionnaire was 0.87. Overall, validation results of the previous studies in the context which reported acceptable levels of reliability and validity indices for the scale, and also the particular reliability measures obtained in the study provided reliable grounds to support the reliability and validity of the scale in this study.

### Resilience questionnaire

One of the most popular scales in assessing learners’ resilience is Ego Resilience scale [73], which is a 14-item questionnaire. In this study, respondents were required to answer the questionnaire on a 6-point Likert scale from 1 (strongly disagree) to 6 (strongly agree). A sample item for resilience is “I quickly get over and recover from being startled”. Learners’ higher scores on the scale point to their greater self-perceived levels of resilience. [73] provided evidence for the validity of the scale in a sample of participants at the age range of 18–23 and its construct validity was confirmed by [74]. In the current attempt, the reliability of the questionnaire was 0.66 through Cronbach’s alpha.

### Academic motivation questionnaire

Academic Motivation Scale [75] including 33 items with two subscales, i.e., Intrinsic Motivation Subscale and the Extrinsic Motivation Subscale, was used. The Intrinsic Motivation Subscale involves three aspects including curiosity (3 items), challenge (9 items), and independent mastery (5 items); while, the Extrinsic Motivation Subscale entails three aspects including pleasing teacher (4 items), dependence on the teacher (6 items), and easy work (6 items). The scale has a five-point Likert scale from completely disagree (score 1) to completely agree (score 5). Some items such (e.g., items 3, 5, 8, and 15) are scored in a reverse order. The lowest and highest scores of this scale are 33 and 165, respectively. Scores of a value between 33 and 66 point to a weak academic motivation level, those of between 66 and 99 refer to an average level of academic motivation, and if the score lies above 99, the level of academic motivation will be at a high level. In [75], the Cronbach’s alpha coefficient of this scale was reported as 0.88 and also in [76] in Iran, it was found to be 0.82. Moreover, [77] confirmed the validity of the scale in an Iranian sample through confirmatory factor analysis. In this research, the Cronbach’s alpha coefficient of the scale was 0.81.

### Data collection procedure

Early in the study, the instructors of the two universities were contacted to invite them participate in the study. Upon their agreement, the researchers provided the consent letters forms along with an online survey for the students. The students were assured that their responses would be kept confidential. Then, they completed the questionnaires, the links of which were shared with them through the well-known WhatsApp application. The collection of data lasted for two months. At the end of this phase, a body of 120 questionnaires were collected.

### Data analysis

Using SPSS software (version 23), both descriptive and inferential statistics were used in this study. First, descriptive statistics were used to check the normality of the obtained data via such indices as Kurtosis and Skewness. Subsequently, Pearson correlation and multiple regressions statistical methods were used to investigate the research hypotheses posed earlier in the study.

## Results

The findings of the present study which include descriptive statistics, the correlation matrix between the research variables, and the results of multiple regression are presented in the following tables.

As the results of Table 1 show, the mean of self-efficacy and resilience variables as predictor variables are 147.76 and 59.92, respectively, and the standard deviations of these variables are 26.4 and 12.51, respectively. Also, the average of academic motivation was 87.28 and its standard deviation was 7.72.

**Table 1:** Descriptive statistics of research variables.

Variables	Mean	SD	Min	Max	N
Self-efficacy	147.76	26.4	64	192	120
Resilience	59.92	12.51	28	84	120
Academic motivation	87.28	7.72	67	106	120

The correlation coefficient between the research variables is reported in Table 2. As shown in this table, there is a positive and significant relationship (r = 0.36) between self-efficacy and academic motivation (p <0.01) and between resilience and academic motivation (r = 0.41).

**Table 2:** Correlation matrix between research variables.

Variables	Self-efficacy	Resilience	Academic motivation
Self-efficacy	1		
Resilience	0.28**	1	
Academic motivation	0.36**	0.41**	1

*Shows the existence of the significant relationship at the level of 0.05.

As shown in Table 3, self-efficacy significantly predicted the students’ academic motivation (p <0.01) with a standard coefficient of 0.026. Also, resilience significantly predicted students’ academic motivation (p <0.01) with a standard coefficient of 0.33. The positive sign of standard coefficients between these variables indicates that increasing learners’ self-efficacy and resilience increases their academic motivation. Also, according to the results of this table, 23% of the variance related to students’ academic motivation is explained by self-efficacy and resilience variables. Therefore, according to the results of Table 3, the research hypotheses posed earlier in this study, i.e., predicting students ’academic motivation based on self-efficacy and resilience variables are accepted. In other words, the results in this investigation showed that both self-efficacy and resilience predicted the students’ academic motivation.

**Table 3:** Results of multiple regression for predicting academic motivation based on self-efficacy and resilience variables.

Variables		Unstandardized Coefficients	Standardized Coefficients					
		B	SE	Β	R	R^2^	t	P
Academic motivation	Constant	63.27	4.13	-			15.30	0.000
	Self-efficacy	0.78	0.025	0.026	0.49	0.23	3.13	0.002
	Resilience	0.209	0.052	0.33			3.92	0.000

## Discussion

The results obtained in this investigation on the significant correlation of self-efficacy and academic motivation and resilience and academic motivation have extended our knowledge of the factors contributing to improved online experience and yield suggestions for future studies. Several reports have established empirical evidence confirming the positive relationships between self-efficacy and language learning (29, 34–37, 39–40). Also, there is some evidence indicating that self-efficacy is a significant factor in online educational contexts [41–43]. In this regard, then, this investigation mirror those of previous studies that had confirmed the importance of students’ self-efficacy in language education, and especially language learning in the online environment. Also, it confirms the association between self-efficacy and academic motivation which was earlier established in the literature [47]. Generally, learners with a higher level of self-efficacy would exert much more learning effort than their counterparts of lower self-efficacy level and can better preserve their optimism. Considering the challenges associated with the online education such as the speed of Internet access, the type of learning device, online test anxiety, and lack of face-to-face interaction, as well as the challenges associated with the pandemic including, task overload, pandemic fears, social isolation, and confinement, those with higher self-efficacy would better accept challenging tasks, retain their interest in class discussions and tasks, and recover from disappointments sooner.

Moreover, research has shown that resilience is a contributing factor in the motivation and proficiency level of language learners [52, 64, 67]. Many studies [68–70] further supported that resilience has a significant role in the ability to manage stress during the COVID-19 pandemic. In this study, the researchers proposed positive relationships between language learners’ academic motivation in online learning and their resilience. This finding can be explained by the SDT which pointed to three factors, i.e., relatedness, autonomy and competence, as the key determinants of both academic motivation and resilience. Resilience, as noted in the literature, can positively affect individuals’ success in various domains. When they face challenges in learning a subject such as a foreign language, their resilience would be triggered to help them overcome the challenges and accordingly their motivation would be enhanced.

The COVID-19 pandemic changed many aspects of individuals’ lives and many contexts, including the university context has been no exception in being affected by the pandemic. Accordingly, normal lives of students changed notably as a consequence of physical distancing measures and also the shift from traditional in-person classes to online learning. Thus, the learners started experiencing a multitude of feelings such as fear, anxiety, and stress and mostly felt less motivated due to the lower social interaction they had. Hence, it is suggested that teachers apply motivation-boosting tasks and activities to prevent the decrease in academic motivation. Two significant factors leading to academic motivation, based on the findings of this investigation, are self-efficacy and resilience. So, language teachers are required to focus on tasks and activities used to enhance self-efficacy, and on educational practices which will reduce insecurity and enhance resilience.

### Implications

Our findings have some implications for teacher training programs. First and foremost, the implication of this study is that every single attempt should be made to reduce unnecessary stressors for learners and promote their resilience. As such, this study suggests that policymakers give an important role to the inclusion of resilience-building tasks and activities by fostering close and respectful connections between students, teachers, and parents. Language teachers can devise tasks and activities that are based on the components of resilience, which would help students have the required resources and competencies to be more self-reliant in learning, creative in problem solving and effective in decision making.

Another significant pedagogical implication which is resulted from the findings of the current attempt is that to improve and upgrade language instruction, language teachers can apply instructional activities which flourish EFL learners’ self-efficacy. Interventions focusing on self-efficacy in educational settings may help come up with a solution to educational problems, but such trainings should be based on careful definition and analysis of self-efficacy components and psychological theory.

## Conclusion

This study confirmed that EFL learners’ self-efficacy and resilience can significantly predict their academic motivation in an online mode of education. Taken together, the findings implied that language teachers need to improve learners’ self-efficacy and resilience in order to assist them in improving their academic motivation. In addition, given that SDT postulates the influence of autonomy, relatedness and competence on learners’ motivation, resilience and self-efficacy, applying strategies to enhance these three basic psychological needs are important for language learners to be able to manage the stressors and challenges associated with learning in both face-to-face and online settings. The results of this research suggest a number of directions for future research. In order to delve into the effect of self-efficacy and resilience on EFL learners’ academic motivation in greater detail, experimental investigations applying activities focusing on these two variables and exploring their effectiveness are clearly of interest.

As with all other studies, the findings of this study are subject to a number of limitations that need to be acknowledged. First, the participant sample was from different language proficiency levels and different universities undergoing various tasks and activities; therefore, studies with homogenized learners are likely to show different results. In this light, further investigations are recommended to replicate these results with learners of the same proficiency level to investigate the possible differences in learners’ self-efficacy and resilience levels. Moreover, this was a correlational study on three variables, namely self-efficacy, resilience and academic motivation with no focus on learners’ language proficiency and academic performance. Therefore, there is abundant room for further progress in exploring and (re)examining the role of self-efficacy and resilience in the second language learning context and its relation to learners’ academic motivation and academic success in online contexts and during the post-COVID pandemic.

## Supporting information

S1 File (RAR)
